# Qinggan Mingshi Granules Inhibited Ferroptosis to Treat Diabetic Retinopathy in Mice Through NRF2/GPX4 Axis

**DOI:** 10.1155/jdr/9978155

**Published:** 2026-01-21

**Authors:** Zhongyong Zhang, Qianqian Jin, Hongmin Zhao, Yingkai Liu, Meng Wang, Zhongqian Han, Jin Wu, Shuquan Lv, Xiaoyun Wang, Wei Chen, Qingjin Wang, Hailong Bai, Yuansong Wang

**Affiliations:** ^1^ Department of Endocrinology, Cangzhou Hospital of Integrated Traditional Chinese Medicine and Western Medicine, Cangzhou, Hebei, China; ^2^ Department of Ophthalmology, Cangzhou Hospital of Integrated Traditional Chinese Medicine and Western Medicine of Hebei, Cangzhou, Hebei, China

**Keywords:** diabetic retinopathy, ferroptosis, lipid peroxidation, NRF2/GPX4 axis, Qinggan Mingshi granules

## Abstract

**Background:**

Diabetic retinopathy (DR) is a common microvascular complication of diabetes, which seriously affected the life quality in diabetic patients. Developing novel therapy to improve DR is essential. Qinggan Mingshi granules (QGMS) have been demonstrated with protective effects on DR clinically. However, the mechanisms of QGMS remain unclear.

**Method:**

In order to more thoroughly investigate the mechanism underlying the positive effects of QGMS on DR, a mouse model of DR was established in this study, and the positive effects of QGMS on the DR mice were observed. Next, the effects of QGMS on ferroptosis and the NRF2/GPX4 axis were investigated. In addition, we used an NRF2 inhibitor to determine whether QGMS inhibits ferroptosis in DR mice via the NRF2/GPX4 axis.

**Result:**

Our results revealed the therapeutic effects of QGMS on DR including improving the permeability of blood–retina barrier (BRB), reducing the pathological changes and ferroptosis in retina. QGMS also induced the expression of NRF2/GPX4 axis in retina. Furthermore, ML385, an NRF2 inhibitor, abolished the effects of QGMS on DR.

**Conclusion:**

This study revealed that QGMS can effectively treat DR by alleviating retinal damage through enhancing the expression of NRF2/GPX4 axis‐related proteins and thus scavenging of LPOs, ultimately reducing ferroptosis.

## 1. Introduction

Diabetic retinopathy (DR) is a common and specific microvascular complication of diabetes mellitus. Approximately one‐third of patients with diabetes worldwide are at risk of developing DR [1]. Epidemiological statistics show that it is projected that by 2030, the number of patients with DR worldwide will increase to over 190 million [2] The progression of DR is associated with vascular abnormalities, impairment of blood–retina barrier (BRB) permeability, progressive microvascular damage and subsequent capillary occlusion, vascular basement membrane (BM) thickening, and abnormalities in the retinal neurons and glia. Symptoms progress from blurred and distorted vision and dark shadows to vitreous hemorrhage, retinal detachment, and ultimately blindness [3]. Unfortunately, reliable DR treatments are limited and mostly aimed at end‐stage disease, with surgical treatments having potentially serious vision‐threatening side effects [4]. Therefore, the discovery of novel agents for the treatment of DR at early stages is essential.

Ferroptosis is a form of cell death caused by the accumulation of iron‐dependent lipid peroxides (LPO) [5]. Excessive irons are cytotoxic and can generate large amounts of reactive oxygen species (ROS) via the Fenton reaction, which in turn oxidizes cell membrane lipids, causing toxic LPO to accumulate in the cell and leading to cell membrane damage, thereby resulting in cell death [6]. Elevated iron levels in the retina have been found to increase LPO and lead to cell death [7]. The ferroptosis inhibitor liproxstatin‐1 has been found to be effective in preventing early DR and maintaining normal visual function [8]. Thus, inhibition of ferroptosis represents a new avenue for research on drugs for DR [9].

There is growing evidence for a potential protective role of NRF2 in the retina. NRF2 agonists increased the expression of antioxidant genes, decreased the expression of inflammatory mediators in the retina, and reduced leukocyte adhesion to retinal vessels after LPS treatment in Nrf2^+/+^ mice, but not in Nrf2^−/−^ mice [10]. Similarly, treatment of wild‐type mice with an NRF2 agonist increased antioxidant gene expression and normalized ischemia–reperfusion‐induced retinal superoxide level; this was not observed in Nrf2^−/−^ mice [11]. In addition, accumulated studies have revealed that the activation of NRF2 significantly inhibits ferroptosis [12].

Qinggan Mingshi granules (QGMS) consist of *Bupleurum chinense DC.*, *Paeonia lactiflora Pall.*, *Rehmannia glutinosa Libosch.*, *Panax notoginseng (Burk.) F.H.Chen*, *Gentiana scabra Bge.*, *Cirsium japonicum DC.*, *Rubia cordifolia L.*, *Chrysanthemum morifolium (Ramat.) Tzvel.*, *Gardenia jasminoides J Ellis*, *Plantago asiatica L.*, *Haliotis diversicolor Reeve*, *Cyathula officinalis Kuan*, *Glycyrrhiza uralensis Fisch.*, and *Lapis lithospermii*, and have been used in the clinical treatment of DR for many years. Clinical studies have shown that QGMS can significantly improve visual acuity and ameliorate macular edema in patients with DR [13]. However, the specific therapeutic mechanism of QGMS has yet to be elucidated. To more thoroughly investigate the mechanism underlying the positive effects of QGMS on DR, a mouse model of DR was established, and the positive effects of QGMS on the DR mice were observed. Next, the effects of QGMS on ferroptosis and the NRF2/GPX4 axis were investigated. In addition, we used an NRF2 inhibitor to determine whether QGMS inhibits ferroptosis in DR mice via the NRF2/GPX4 axis.

## 2. Methods

### 2.1. Animals and Materials

Male C57BL/6 mice (aged 6–8 weeks, 20–22 g) were purchased from Sibeifu Biotechnique Co. Ltd. QGMS was obtained from Cangzhou Hospital of Integrated Traditional Chinese Medicine and Western Medicine. The main components are shown in Figure S1, and the detailed elution gradient is provided in Table S1. Other reagents and kits were included in the Supporting information.

### 2.2. Animal Experiments

All animals were housed in an environment with access to food and water *ad libitum* and reared in an artificial 12:12 hour light‐dark cycle, a constant temperature of 23 ± 2°C, and relative humidity of 60 ± 5%. The mouse DR model was established with slight changes as previously described [14]. After acclimatization for 1 week, mice in the DR group were fed a high‐fat, high‐sugar diet (HFD) for 8 weeks, while mice in the normal control (NC) group were fed a normal diet. At the end of week 8, mice fed the HFD were administered an intraperitoneal injection of 45 mg/kg STZ for 5 consecutive days, while mice in the NC group were injected with the same volume of vehicle. The establishment of the diabetic mouse model was considered successful when blood glucose ≥ 16.7 mmol/L [15]. All experiments were approved by Ethics Committee of Cangzhou Hospital of Integrated Traditional Chinese Medicine and Western Medicine of Hebei (approval no. CZX2025‐KY‐025).

The therapeutic effects of QGMS in DR mice and the effects on retinal ferroptosis and the NRF2/GPX4 axis were investigated. A total of 120 mice were randomly divided into NC, DR, calcium dobesilate (CaD), low‐dose (QGMS‐L), medium‐dose (QGMS‐M), and high‐dose QGMS (QGMS‐H) groups. The DR model was established in all groups except for the NC group. After establishing the model, the CaD group was administered CaD 0.25 g/kg/d by gavage [16]. The QGMS‐L, QGMS‐M, and QGMS‐H groups were administered 1.2, 2.4, and 4.8 g/kg/d QGMS by gavage, respectively. The drug dosages were determined based on the body weight of rats and the normal clinical usage of QGMS [17]. The medium dose was considered as the equivalent dose, with the low dose being 0.5 times the medium dose and the high dose being twice the medium dose. In both the NC and DR groups, equal volumes of vehicle were administered by gavage daily. All mice were treated continuously for 8 weeks. The QGMS‐M dose was the equivalent human dose based on body surface area dosing.

In evaluating the therapeutic effects of QGMS on DR and the effects on retinal ferroptosis and NRF2/GPX4 axis following NRF2 inhibitor administration, 100 mice were randomly divided into NC, DR, Ferrostatin‐1 (Fer‐1), QGMS, and QGMS+ML385 groups. The DR model was established in all groups except for the NC group. After model establishment, the Fer‐1 group was intraperitoneal injected with 1 mg/kg of Fer‐1 and vehicle daily by gavage [18]. The QGMS group was administered vehicle intraperitoneally and 4.8 g/kg of QGMS daily by gavage. The QGMS + ML385 group was injected with 30 mg/kg of ML385 intraperitoneally [19] and 4.8 g/kg of QGMS daily by gavage. Equal volumes of vehicle were administered daily by intraperitoneal injection and gavage in both the NC and DR groups. All mice were treated continuously for 8 weeks.

During treatment, mice were weighed every 2 weeks and fasting blood glucose (FBG) was tested. At the end of the 8‐week treatment, 6 mice per group were randomly selected for the Evans blue (EB) leakage assay.

**Figure 1:**
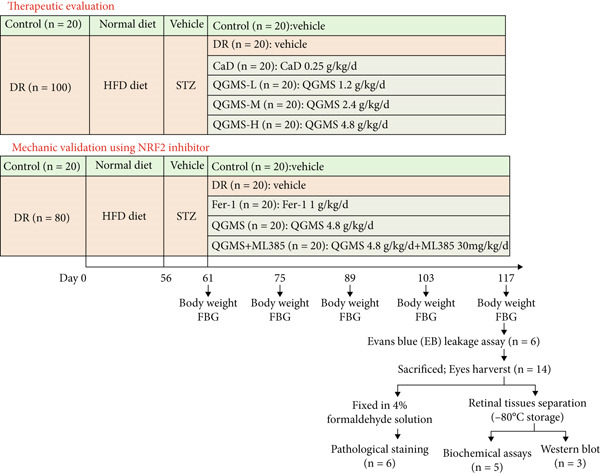
Experimental design of this study.

Subsequently, mice were anesthetized with sodium pentobarbital (50 mg/kg), blood samples were collected from the abdominal aorta, and serum was obtained through centrifugation. The mice were then euthanized by an overdose of sodium pentobarbital. Besides, both eyes were harvested in each group and eyes in six mice were fixed in 4% paraformaldehyde solution for pathological and TUNEL staining, eyes in five mice per group were used for biochemical assays, the remaining three mouse eyes were used for western blot (Figure 1).

### 2.3. EB Leakage Assay

The EB leakage assay for detecting vascular permeability in the mouse retina were performed as described previously [20, 21]. Briefly, a phosphate‐buffered saline (PBS) solution of 2% EB (10 *μ*L/g body weight) was injected intravenously. After circulation of the dye for 2 h, the thorax was opened and the mice were perfused with PBS through the left ventricle for 30 min until the fluid in the right atrium became colorless. After perfusion, the retinas were removed, dried, and weighed. Each retina was then immersed in 150 *μ*L of formamide and incubated at 70°C for 18 h to extract the dye. The extract was ultracentrifuged at 10,000 × g for 45 min. The absorbance at 620 nm of the supernatant was measured. The concentration of dye in the extract was calculated using the standard curve of EB in formamide. Disruption of the BRB was calculated using the following equation: EB (*μ*g)/retina dry weight (g).

### 2.4. HE and TUNEL Staining

After the eyes were fixed in 4% formaldehyde solution, the left and right eyes were used to make 5 *μ*m paraffin sections and retinal flat‐mount preparations, respectively. After routine hematoxylin‐eosin (HE) staining of paraffin sections, pathological changes in the retinal layers were observed under light microscopy, and the retinal thickness was calculated [22]. Paraffin sections were stained with TUNEL to observe cell damage and death and imaged under fluorescence microscopy. Aerosol optical depth (AOD) values were calculated using ImageJ software.

### 2.5. Detection of Ferroptosis and NRF2/GPX4 Axis‐Related Factors

Fresh retinal tissues were collected from each group of mice. The levels of 4‐hydroxynonenal (4‐HNE), malondialdehyde (MDA), ROS, total iron, total glutathione (GSH), and glutathione disulfide (GSSG) in the retina of each group were measured per the manufacturers’ instructions of the respective detection kits. Among them, MDA, total iron, and GSH/GSSG were detected using biochemical colorimetric assays, 4‐HNE was detected using ELISA, and ROS was detected using a fluorescent probe.

### 2.6. Western Blot

Total protein was extracted from fresh retinal tissues collected from each group of mice. Protein concentration was determined, and proteins were fully denatured by heating at 99°C for 5 min after addition of protein loading buffer. The proteins in each sample were separated by SDS‐PAGE electrophoresis and transferred onto a PVDF membrane. After blocking with 5% skim milk for 1 h, primary antibodies against the target proteins were added and incubated at 4°C overnight. The membranes were washed, and secondary antibodies were added and incubated at room temperature for 1 h. The membranes were washed again, and ECL reagent was added and exposed using an automatic gel imaging system to obtain protein bands. The grayscale values of the protein bands were analyzed using ImageJ software, and relative expression levels were calculated.

### 2.7. Statistics

SPSS 26.0 software was used for statistical analysis. Results were expressed as mean ± standard deviation (SD). Comparisons between multiple groups were made using one‐way analysis of variance (ANOVA) with the Tukey‐HSD post hoc test. Differences with *p* < 0.05 were considered statistically significant.

## 3. Results

### 3.1. Therapeutic Effects of QGMS in DR Mice

Changes in body weight and FBG of mice in each group were dynamically observed every 2 weeks from the start of drug administration. At the end of 8 weeks of treatment, the DR group exhibited a significant decrease in body weight and a significant increase in FBG compared with the NC group. Compared with the DR group, QFMS treatment improved body weight loss and hyperglycemia (Figure 2a,b), as well as insulin resistance (Figure S2). However, CaD treatment did not improve the body weight loss, hyperglycemia, and insulin resistance in DR mice. Significantly, QGMS treatment had no significant effect on uptake in DR mice (Figure S3a), suggesting that the improvements in body weight and blood glucose were independent of alterations in uptake. EB leakage assay results showed significantly more dye leakage in the eyes of mice in the DR group than in the NC group, suggesting disruption of the BRB. Dye leakage was significantly reduced in both the CaD and QGMS groups, suggesting amelioration of BRB disruption (Figure 2c). HE staining showed increased retinal thickness and disorganized retinal layers of mice in the DR group compared with that of the NC group, suggesting the presence of edema in the retina; this was improved to varying degrees after treatment with CaD or QGMS (Figure 2d,e). In addition, these treatment effects of QGMS exhibited dose dependence and did not differ significantly between the high‐dose and CaD groups.

Figure 2QGMS treatment ameliorated DR in mice. Mice received HFD for 8 weeks and STZ injection (45 mg/kg) for 5 consecutive days to induce DR. Then, DR mice were received different dose of QGMS treatment. CaD (0.25 g/kg) was used as the positive control drug. All drugs were treated orally per day for 8 weeks. The body weight and FBG were observed every 2 weeks during QGMS treatment. Besides, EB leakage assay was conducted to evaluate the permeability of BRB. HE staining was also used to observe the pathological changes in retina. (a, b) QGMS treatment did not affect the weight (a) and FBG (b) levels in DR mice (*n* = 20 per group). (c) EB leakage assay showed that QGMS treatment improved the dysfunction of BRB (*n* = 6 per group). (d, e) HE staining indicated that QGMS reduced the pathological changes in retina (400×, *n* = 6 per group). ##*p* < 0.01 compared with NC; ∗*p* < 0.05, ∗∗*p* < 0.01 compared with DR group.

**Figure:**
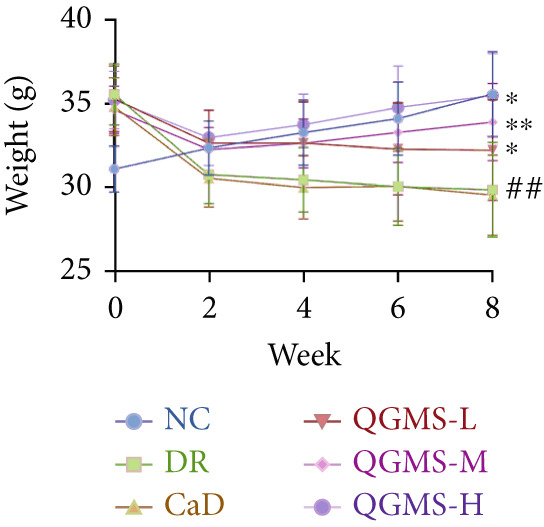
(a)

**Figure:**
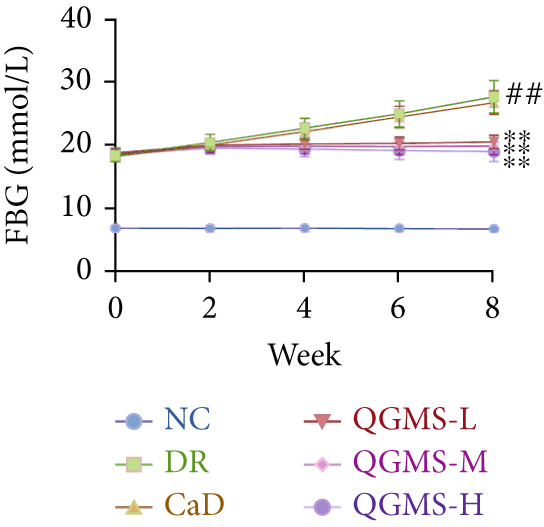
(b)

**Figure:**
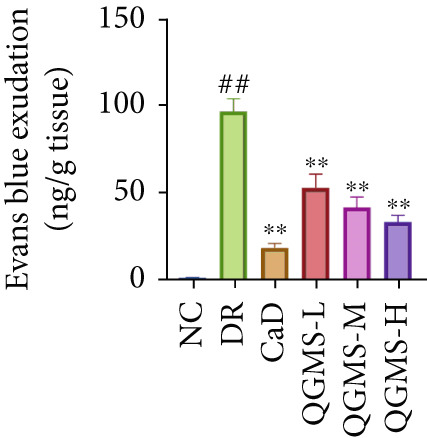
(c)

**Figure:**
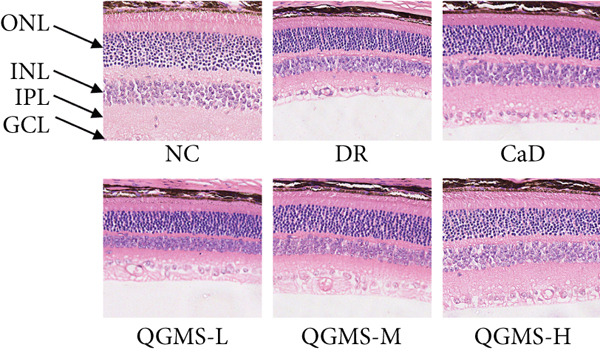
(d)

**Figure:**
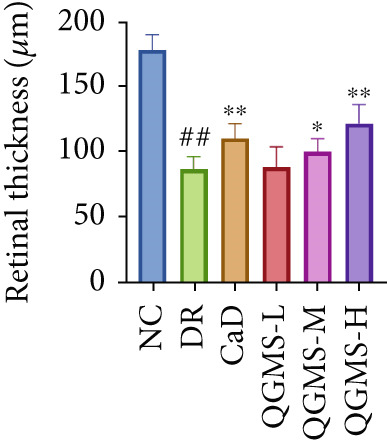
(e)

### 3.2. Effect of QGMS on Retinal Ferroptosis and the NRF2/GPX4 Axis in DR Mice

TUNEL staining indicated different degrees of retinal cell damage and death in the DR mice compared with the NC group. This damage was significantly ameliorated by either CaD or QGMS treatment (Figure 3a,b). In addition, total iron levels were significantly elevated in the retinas of the DR mice, which was similarly reduced by CaD and QGMS treatment (Figure 3c). Detection of lipid peroxidation‐related indicators revealed higher levels of ROS (Figure 3d), 4‐HNE (Figure 3e), and MDA (Figure 3f) in the retina of DR mice than in the NC group, whereas both CaD and QGMS reduced these indicators to various degrees. These results suggest severe ferroptosis in the retina of DR mice, whereas treatment with CaD and QGMS protects the retina and reduces ferroptosis.

Figure 3QGMS treatment reduced ferroptosis in retina. Cell death in retina in DR mice was observed by TUNEL staining. The positive area of TUNEL staining was quantified using ImageJ. Besides, the levels of total iron, and the levels of LPO‐related factors (ROS, 4‐HNE and MDA) in retina were tested using relative kits to evaluate the effects of QGMS on ferroptosis. (a, b) TUNEL staining showed that QGMS ameliorated cell death in retina (200×, *n* = 6 per group). (c) QGMS decreased the total iron levels in retina (*n* = 5 per group). (d–f) QGMS decreased the levels of ROS (d), 4‐HNE (e) and MDA (f) in retina (*n* = 5 per group). ##*p* < 0.01 compared with NC; ∗*p* < 0.05, ∗∗*p* < 0.01 compared with DR group.

**Figure:**
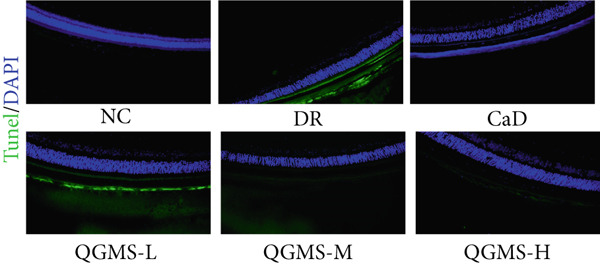
(a)

**Figure:**
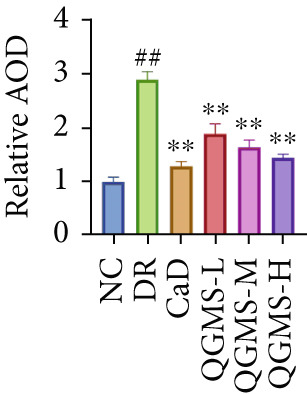
(b)

**Figure:**
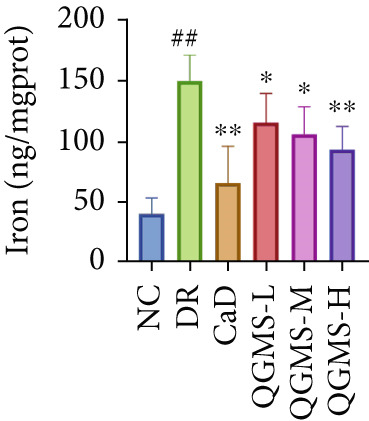
(c)

**Figure:**
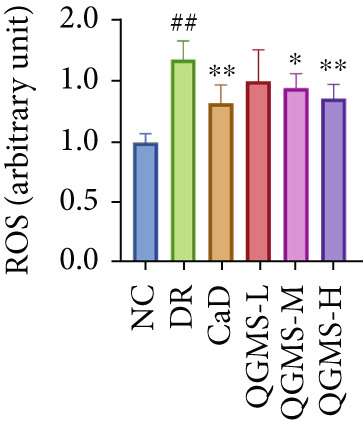
(d)

**Figure:**
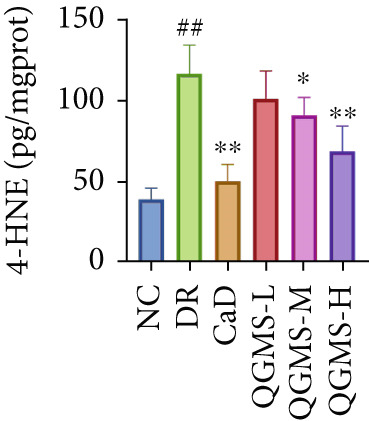
(e)

**Figure:**
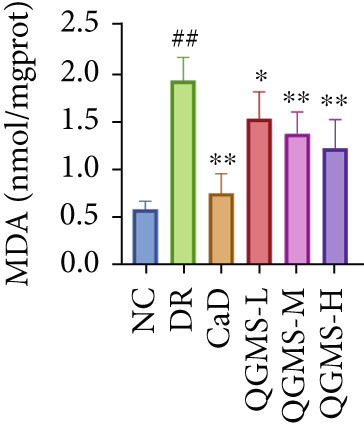
(f)

Detection of NRF2/GPX4 axis‐associated factors showed a significantly lower GSH/GSSG ratio inside the retina in the DR group than in the NC group, indicating increased GSH depletion (Figures 4a, 4b, and 4c). Western blot revealed significantly reduced levels of the NRF2/GPX4 axis‐related proteins NRF2, SLC7A11, FTL, and GPX4 in the retinas of mice in the DR group, suggesting inhibition of the NRF2/GPX4 axis (Figures 4d, 4e, 4f, 4g, and 4h). After CaD or QGMS treatment, the GSH/GSSG ratio increased and the NRF2/GPX4 axis‐associated proteins improved to varying degrees, suggesting that both CaD and QGMS can enhance the NRF2/GPX4 axis.

Figure 4QGMS treatment induced the expression of NRF2/GPX4 axis‐related factors in retina. The levels of GSH and GSSG in retina were tested using kits and the ratio of GSH/GSSG was calculated. The expression of NRF2/GPX4 axis‐related proteins (NRF2, SLC7A11, FTL and GPX4) was investigated using western blot and the results were quantified using Image J. (a–c) QGMS treatment increased the ratio of GSH/GSSH in retina (*n* = 5 per group). (d–h) QGMS treatment increased the expression of NRF2 (d, e), SLC7A11 (d, f), FTL (d, g) and GPX4 (d, h) in retina (*n* = 3 per group). ##*p* < 0.01 compared with NC; ∗*p* < 0.05, ∗∗*p* < 0.01 compared with DR group.

**Figure:**
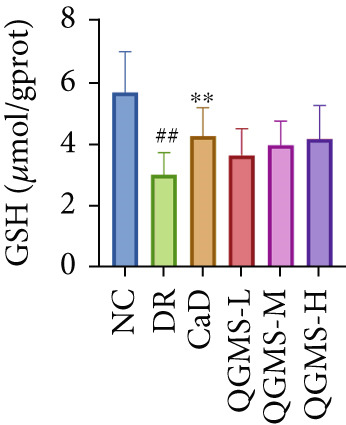
(a)

**Figure:**
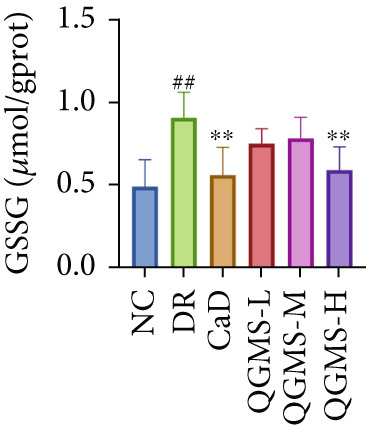
(b)

**Figure:**
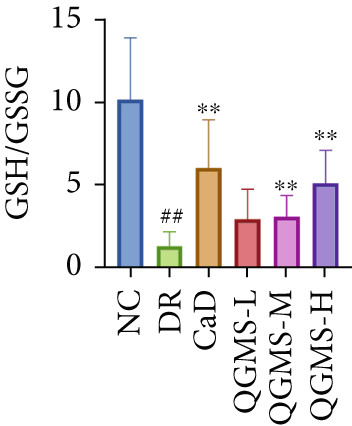
(c)

**Figure:**
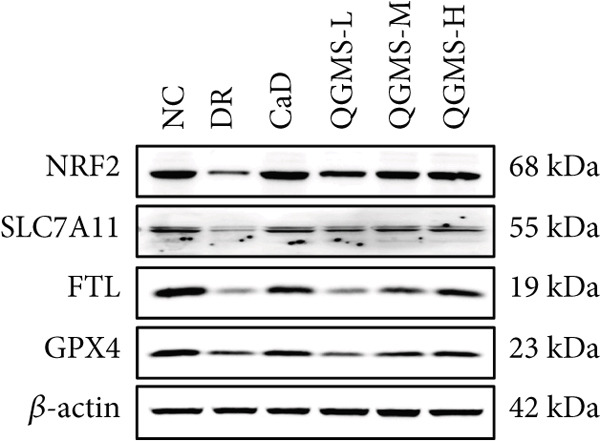
(d)

**Figure:**
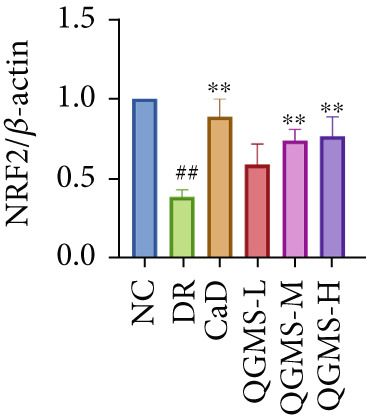
(e)

**Figure:**
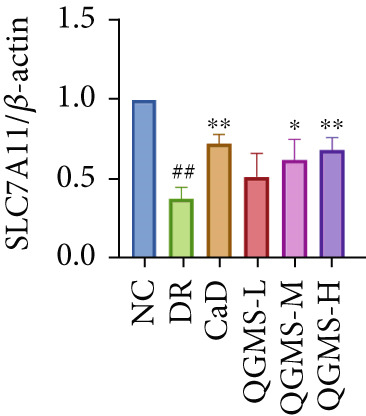
(f)

**Figure:**
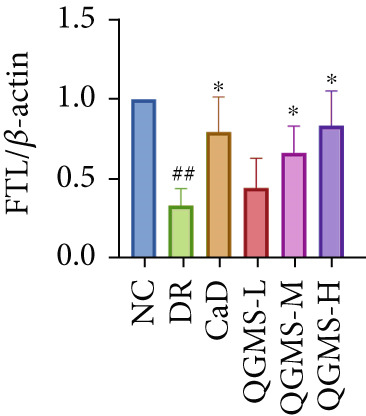
(g)

**Figure:**
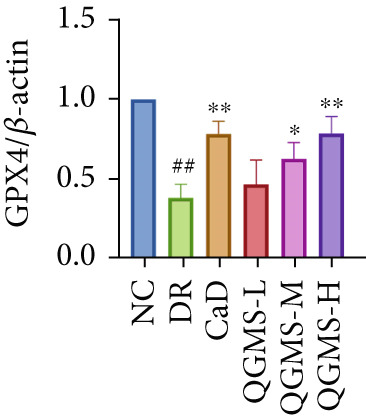
(h)

The above results demonstrate that QGMS can treat DR in a dose dependent manner and that there is no significant difference in therapeutic efficacy between QGMS‐H and CaD. We therefore selected the QGMS‐H dose for subsequent validation testing.

### 3.3. ML385, a NRF2 Inhibitor, Eliminates the Therapeutic Effect of QGMS in DR Mice

Next, we used the ferroptosis inhibitor Fer‐1 and the NRF2 inhibitor ML385 to compare the efficacy of QGMS with Fer‐1 in alleviating DR and to evaluate changes in the therapeutic efficacy of QGMS in DR mice after inhibition of NRF2.

Changes in body weight and FBG of mice in each group were dynamically observed every 2 weeks from the start of drug administration. At the end of the 8‐week treatment, body weight was significantly decreased and FBG was significantly increased in the DR group compared with the NC group. Mice in the Fer‐1 and QGMS group had significantly increased body weight and significantly decreased FBG compared with the DR group, whereas there was no significant difference among the QGMS + ML385 and DR groups (Figures 5a,b). Meanwhile, there were no significant differences in intake among the groups (Figure S3b), suggesting that these indicators were independent of intake. EB leakage assay results showed significantly more dye leakage in the eyes of mice in the DR group than in the NC group. Both the Fer‐1 and QGMS groups had significantly reduced dye leakage, but the QGMS + ML385 group did not exhibit significantly reduced dye leakage (Figure 5c). HE staining showed a disorganized arrangement of retinal layers in the DR group, and retinal thickness was significantly increased compared with the NC group, suggesting retinal edema. Both Fer‐1 and QGMS treatments improved these histopathological findings, but the histopathology in the QGMS + ML385 group was similar to that of the DR group. In addition, these therapeutic effects of QGMS did not differ significantly from those of Fer‐1 (Figure 5d,e). These results indicate that the therapeutic effect of QGMS on DR mice can be abolished by ML385.

Figure 5NRF2 inhibitor abolished the therapeutic effects of QGMS on DR mice. DR mice received intraperitoneal injection of ML385 (30 mg/kg), a NRF2 inhibitor, to inhibit NRF2 activation followed by QGMS treatment (4.8 g/kg). The effects of QGMS on DR mice before and after NRF2 inhibition were observed. Furthermore, DR mice received intraperitoneal injection of Fer‐1 (1 mg/kg) to inhibit ferroptosis in order to compare the effects between QGMS and ferroptosis inhibitor. (a, b) The body weight loss (a) and hyperglycemia (b) in DR mice was not alleviated by QGMS and QGMS+ML385 treatment, whereas Fer‐1 ameliorated the body weight loss and hyperglycemia in DR mice (*n* = 20 per group). (c) EB leakage assay showed that the ameliorative effects of QGMS on BRB dysfunction were abolished following NRF2 inhibition (*n* = 6 per group). (d, e) HE staining showed that NRF2 inhibition abolished the effects of QGMS on pathological changes in retina (400×, *n* = 6 per group). ##*p* < 0.01 compared with NC; ∗∗*p* < 0.01 compared with DR group.

**Figure:**
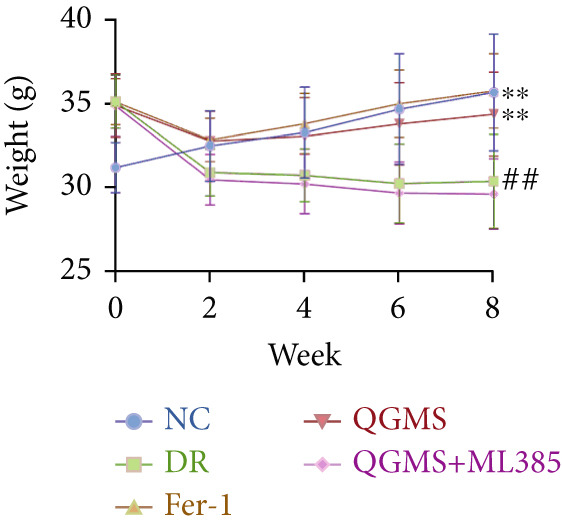
(a)

**Figure:**
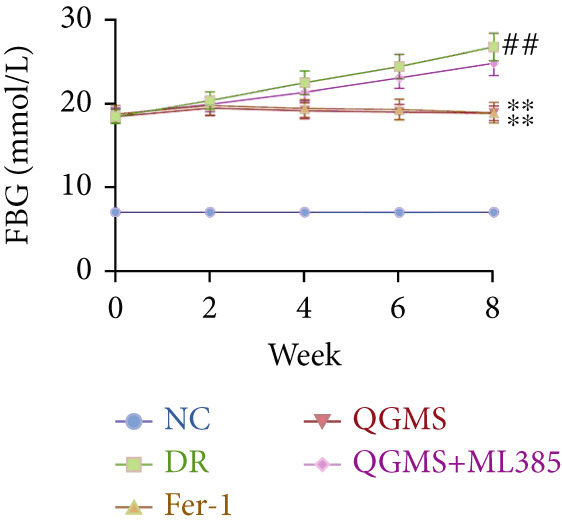
(b)

**Figure:**
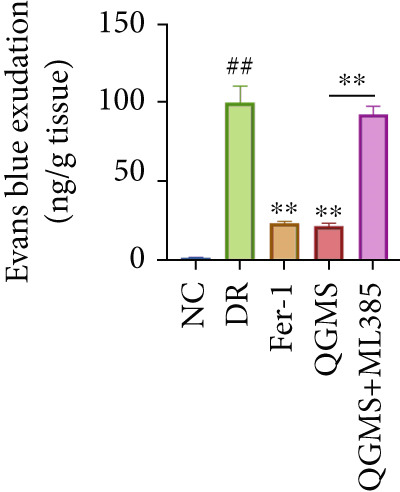
(c)

**Figure:**
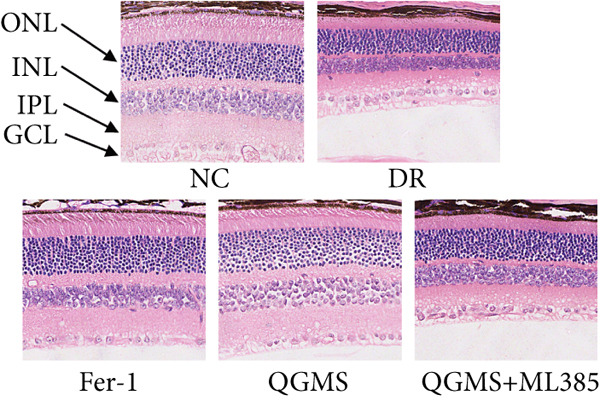
(d)

**Figure:**
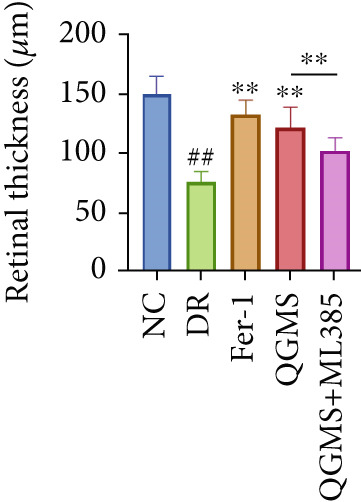
(e)

### 3.4. ML385 Abolishes the Effects of QGMS on Retinal Ferroptosis and the NRF2/GPX4 Axis in DR Mice

TUNEL staining indicated different degrees of retinal cell damage and death in the DR mice compared with the NC group (Figure 6a,b). In addition, total iron levels were significantly elevated in the retinas of the DR mice (Figure 6c). Detection of lipid peroxidation‐related indicators revealed significantly elevated levels of ROS (Figure 6d), 4‐HNE (Figure 6e), and MDA (Figure 6f) in the retina of DR mice compared with the NC group. However, both Fer‐1 and QGMS treatments improved these indicators to varying degrees, and there were no significant differences between the effects of the two therapeutic agents. The QGMS + ML385 group did not differ significantly from the DR group. These results indicate that the therapeutic effects of QGMS on retinal ferroptosis in DR mice were abolished by ML385.

Figure 6NRF2 inhibitor abolished the inhibitory effects of QGMS on ferroptosis. Cell death in retina in DR mice following QGMS and ML385 treatment was observed by TUNEL staining. The positive area of TUNEL staining in each group was quantified using ImageJ. Besides, the levels of total iron, and the levels of LPO‐related factors (ROS, 4‐HNE and MDA) in retina in DR mice following QGMS and ML385 treatment were tested using relative kits to evaluate the effects of QGMS on ferroptosis after NRF2 inhibition. (a, b) TUNEL staining showed that NRF2 inhibitor abolished the ameliorative effects of QGMS on cell death in retina (200×, *n* = 6 per group). (c) QGMS treatment did not affect the total iron levels in retina following NRF2 inhibitor treatment (*n* = 5 per group). (d–f) QGMS treatment did not affect the levels of ROS (d), 4‐HNE (e) and MDA (f) in retina following NRF2 inhibition (*n* = 5 per group). ##*p* < 0.01 compared with NC; ∗*p* < 0.05, ∗∗*p* < 0.01 compared with DR group.

**Figure:**
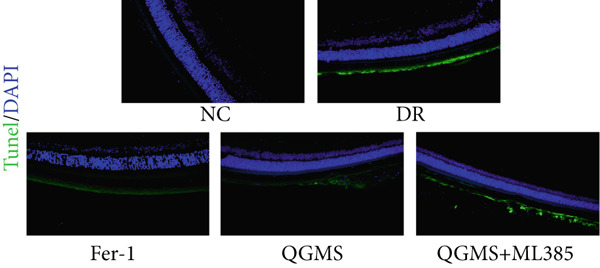
(a)

**Figure:**
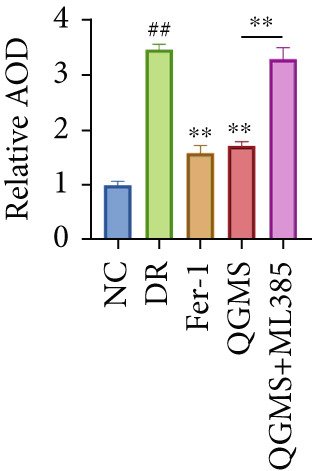
(b)

**Figure:**
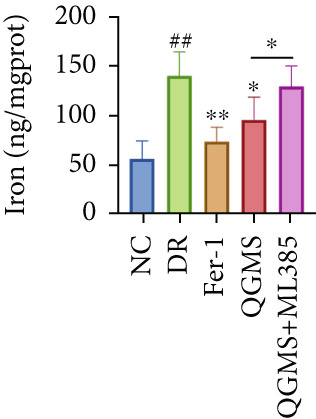
(c)

**Figure:**
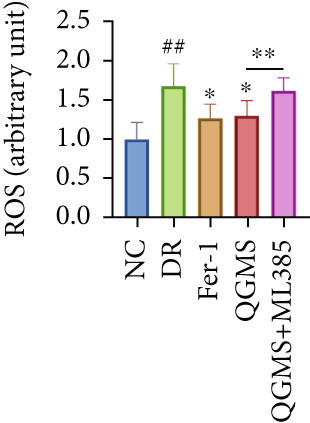
(d)

**Figure:**
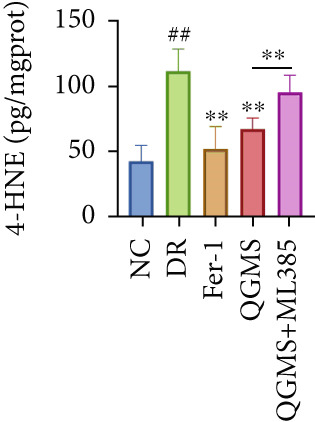
(e)

**Figure:**
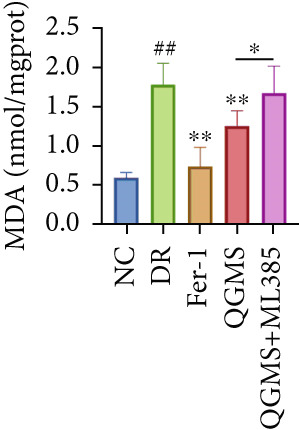
(f)

Detection of NRF2/GPX4 axis‐associated factors showed a significantly decreased GSH/GSSG ratio inside the retina in the DR group compared with the NC group (Figures 7a, 7b, and 7c). Western blot revealed significantly reduced levels of the NRF2/GPX4 axis‐related proteins NRF2, SLC7A11, FTL, and GPX4 in the retina of mice in the DR group (Figures 7d, 7e, 7f, 7g, and 7h). After treatment with Fer‐1 or QGMS, these changes improved to varying degrees; there was no significant difference between Fer‐1 and QGMS. On the other hand, the QGMS + ML385 group did not differ from the DR group, indicating that ML385 abrogates the enhancement of the NRF2/GPX4 axis by QGMS.

Figure 7NRF2 inhibitor abolished the effects of QGMS on NRF2/GPX4 axis. The levels of GSH and GSSG in retina in DR mice after QGMS and ML385 treatment were tested using kits and the ratio of GSH/GSSG was calculated. The expression of NRF2/GPX4 axis‐related proteins (NRF2, SLC7A11, FTL and GPX4) was investigated using western blot and the results were quantified using Image J in order to evaluated the effects of QGMS on NRF2/GPX4 axis following NRF2 inhibition. (a–c) QGMS treatment did not affect the ratio of GSH/GSSH in retina after NRF2 inhibition (*n* = 5 per group). (d–h) QGMS treatment did not affect the expression of NRF2 (d, e), SLC7A11 (d, f), FTL (d, g) and GPX4 (d, h) in retina after treatment with NRF2 inhibitor (*n* = 3 per group). ##*p* < 0.01 compared with NC; ∗*p* < 0.05, ∗∗*p* < 0.01 compared with DR group.

**Figure:**
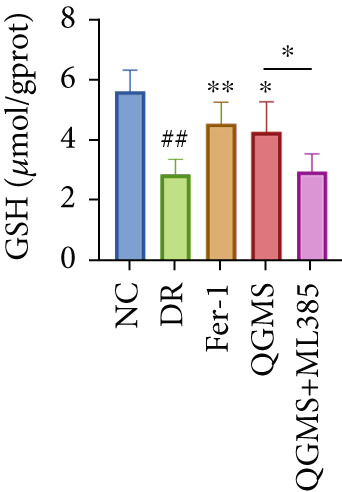
(a)

**Figure:**
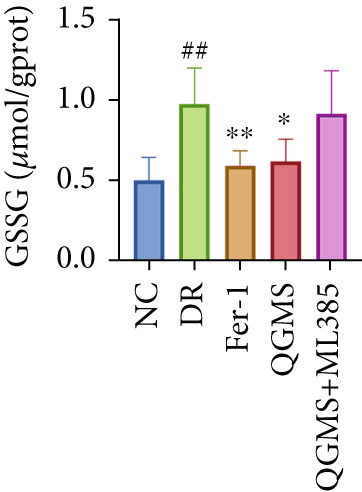
(b)

**Figure:**
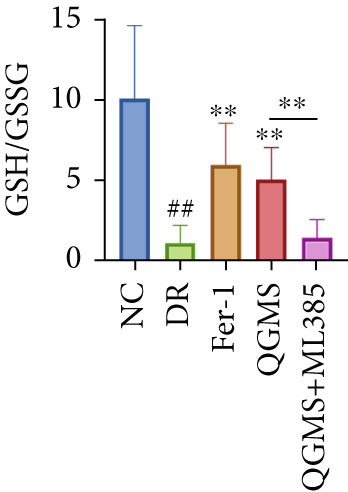
(c)

**Figure:**
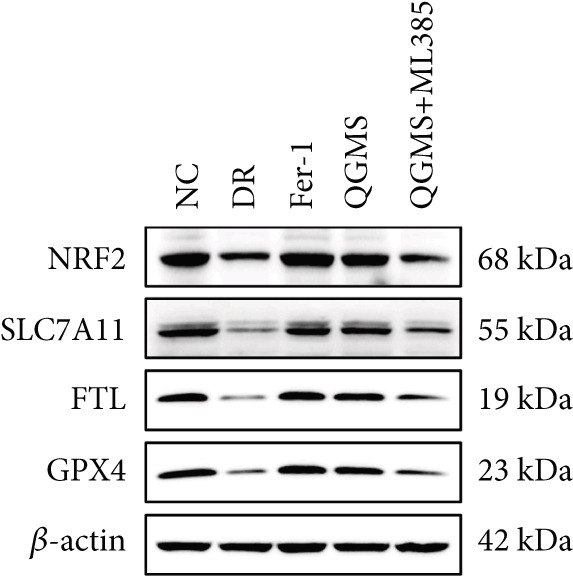
(d)

**Figure:**
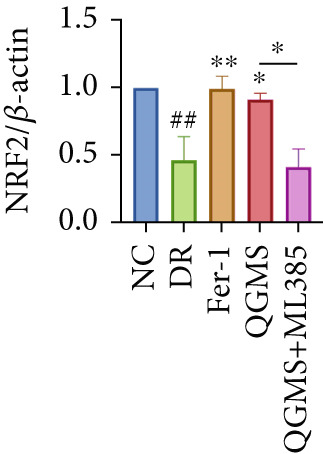
(e)

**Figure:**
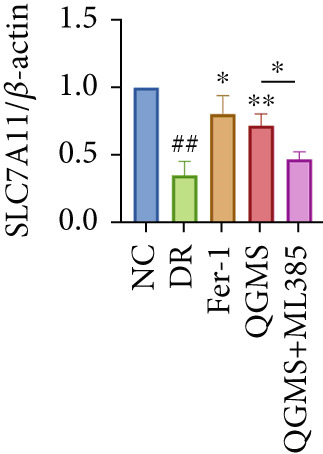
(f)

**Figure:**
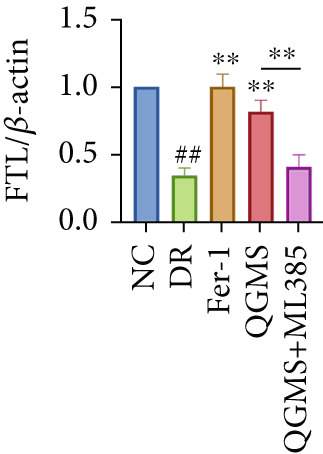
(g)

**Figure:**
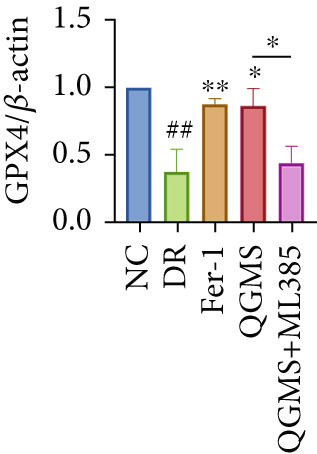
(h)

These results further demonstrate the efficacy of QGMS in reducing ferroptosis in the treatment of DR by enhancing the NRF2/GPX4 axis.

## 4. Discussion

Retinal vasculopathy is the most fundamental pathological change in DR. The retinal vasculature is a multicellular structure composed of endothelial cells, pericytes, and vascular smooth muscle cells closely associated with macroglia, microglia, and neurons. This structure responds dynamically to complex circulatory and neural signals to control blood flow and regulate the BRB. Diabetes significantly alters these behaviors [23, 24]. The mouse DR model is a common small‐animal model used in DR research that faithfully reproduces the early lesions of DR, including retinal capillary BM thickening, endothelial cell damage with pericyte loss, increased vascular permeability, and capillary non‐perfusion [25]. Among these, BM thickening is a hallmark lesion of DR that leads to impaired endothelial cell–pericyte communication as well as faulty retinal capillary autoregulation [26, 27]. When endothelial cells die, retinal capillaries become acellular. This vascular degeneration is a foundation of the progressive ischemia in DR and is commonly found in long‐term diabetic animal models and specimens from patients with DR [28]. Following vascular endothelial injury, the BRB collapses rapidly in a relatively short period of time [29, 30]. On the other hand, peripapillary retinal cell loss is a hallmark of early DR and can occur 2 months after development of diabetes, whereas the appearance of acellular capillaries usually occurs after 6 months of disease [31]. Pericyte loss weakens the BRB and leads to capillary instability and vascular leakage. The BRB is formed by tight junctions between neighboring cells and controls the entry of proteins and other macromolecules from the blood into the retina. It can effectively limit the permeability of blood vessels adjacent to cells, thereby preventing the transport of substances between cells [32]. BRB collapse leads to leakage of macromolecules as well as neuronal and glial swelling, further exacerbating macular edema and promoting end‐stage DR [33]. Accumulation of advanced glycation end products in patients with diabetes has been found to result in cross‐linking of the vascular matrix, resulting in protein degradation and loss of vascular elasticity, which in turn affects vascular cell viability [34, 35]. Clinical evidence clearly demonstrates that good glycemic control in the early stages of diabetes reduces the development and progression of DR and other vascular complications [36]. In the present study, we found that QGMS significantly improved diabetic symptoms in DR mice, including improving body weight and blood glucose. In addition, examination of the retina revealed a dramatic improvement in leakage due to BRB collapse, including decreased retinal thickness, indicating reduced edema, and a reduced number of acellular capillaries, which directly indicates improvement of DR. These results demonstrate that QGMS exerts significant therapeutic effects on retinopathy in DR mice. In addition, CaD was used as a positive‐control drug in the study. CaD has been widely shown to be effective in treating DR at both the systemic level and locally in the fundus [37, 38]. However, the side effects of CaD have been reported in various aspects, such as causing tachycardia, nausea, and diarrhea [39]. The QGMS‐H group did not significantly differ from the CaD group, suggesting that QGMS may serve as an alternative therapeutic agent for DR. Nevertheless, further research is required to evaluate the therapeutic safety advantages of QGMS, in order to provide more detailed experimental evidences for the usage of QGMS in clinic.

Although other diabetes‐related biochemical responses have been associated with DR, such as increased aldose reductase activity, oxidative stress, nitrosylation, and activation of the hexosamine pathway, overproduction of peroxides has been suggested to be a common thread between these seemingly separate pathways [40]. We therefore hypothesized that ferroptosis, which is closely related to peroxide production, plays an important role in the ameliorative effects of QGMS on DR. In addition to the Fenton reaction, which releases large amounts of ROS, high levels of ROS are themselves capable of causing localized tissue and cellular hypoxia. In turn, long‐term chronic hypoxia exacerbates oxidative stress damage to cells, releasing even more ROS [9]. ROS react with membrane phospholipids, enzymes, polyunsaturated fatty acid side chains, and nucleic acids to form LPOs such as MDA and 4‐HNE via lipid peroxidation. Accumulated LPOs eventually alter the fluidity and permeability of the cell membrane, disrupting cellular structure and function [41]. In the present study, we found that QGMS significantly ameliorated retinal cell damage and reduced total iron levels in DR mice. In addition, QGMS reduced the levels of ROS and the LPOs MDA and 4‐HNE in the retina, suggesting that LPO was alleviated. Similarly, the QGMS‐H group differs significantly from the CaD group, further suggesting the efficacy of QGMS in the treatment of DR.

GPX4 is the only known enzyme capable of directly scavenging LPOs and plays a co‐regulatory role in the inhibition of ferroptosis [42]. Cystine is imported into the cell by system xCT, composed of SLC7A11 and SLC3A2, and undergoes a series of reactions to form GSH, a substrate that relieves the cytotoxicity of LPOs in addition to GPX4 [43, 44]. NRF2 is a redox‐sensitive essential leucine zipper region transcription factor believed to be a component of the most important cellular pathway protecting cells from oxidative stress [45, 46]. The entry of NRF2 into the nucleus and binding of NRF2 to antioxidant response element (ARE) sequences activates the expression of a variety of antioxidant genes, including GPX4. This is the basis of the NRF2‐ARE defense pathway, which plays an important role in protecting the retinal vascular system from ROS‐related injury [47–49]. Studies have shown that a hyperglycemic environment inhibits the NRF2 pathway in retinal pigment epithelial cells, leading to reduced antioxidant capacity. The principal mechanism is the inhibition of molecule downstream of NRF2, such as GPX4 [50]. The results of the present study showed that QGMS significantly elevated the GSH/GSSG ratio and ameliorated GSH depletion in the retinal tissue of DR mice. Meanwhile, the levels of the NRF2/GPX4 axis‐related proteins NRF2, SLC7A11, FTL, and GPX4 were significantly elevated. Research has reported that high NRF2 protein expression implying a greater amount of NRF2 entering the nucleus [51]. These results suggested that QGMS enhances the inhibited NRF2/GPX4 axis in the retina of DR mice. Future studies should employ techniques such as immunofluorescence and dual‐luciferase assays to directly observe the nuclear translocation level of NRF2, providing references for researchers investigating the mechanistic actions of NRF2. Concurrently, there was no significant difference between the effects of QGMS‐H and CaD on the NRF2/GPX4 axis. We therefore selected the QGMS‐H group for subsequent validation tests.

To further elucidate the mechanism by which QGMS ameliorates retinal damage in DR mice, we introduced the ferroptosis inhibitor Fer‐1 and the NRF2 inhibitor ML385 to investigate changes in the therapeutic effects of QGMS after inhibition of NRF2 in DR mice. Fer‐1 significantly reduces ferroptosis in photoreceptor cells exposed to light, including iron overload, GSH depletion, increased MDA, and decreased SLC7A11 and GPX4 protein expression [52]. ML385 is a common inhibitor of the NRF2 pathway. Studies have shown that the therapeutic effects of chlorogenic acid on BRB damage can be reversed by ML385 [21]. The results of the present study showed that Fer‐1 could treat diabetic symptoms and alleviate retinal damage in DR mice, as well as reduce ferroptosis and enhance the NRF2/GPX4 axis. There was no significant difference between the effects of Fer‐1 and QGMS. However, ML385 abolished the therapeutic effects of QGMS, demonstrating that QGMS alleviates ferroptosis primarily by enhancing the NRF2/GPX4 axis.

## 5. Conclusion

**Figure 8:**
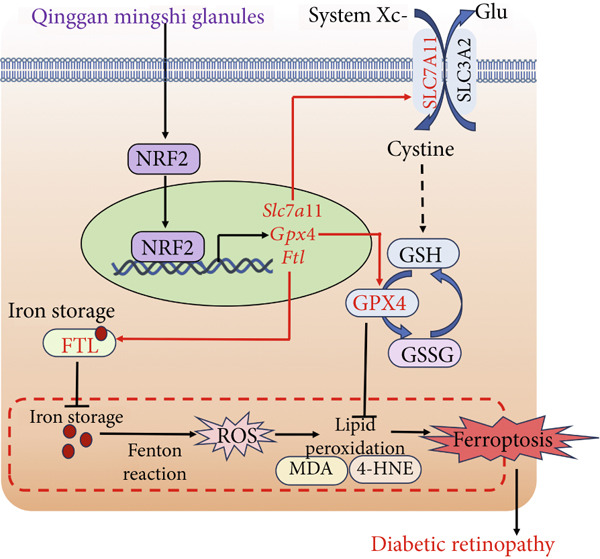
Overview of major findings in this study. QGMS can effectively treat DR by alleviating retinal damage through enhancing the expression of NRF2/GPX4 axis‐related proteins and thus scavenging of LPOs, ultimately reducing ferroptosis.

In summary, QGMS can effectively treat DR by alleviating retinal damage through enhancing the expression of NRF2/GPX4 axis‐related proteins and thus scavenging of LPOs, ultimately reducing ferroptosis (Figure 8).

## Ethics Statement

Animal experiments were approved by Ethics Committee of Cangzhou Hospital of Integrated Traditional Chinese Medicine and Western Medicine of Hebei (Approval no. CZX2025‐KY‐025).

## Disclosure

All authors approved the submitted version.

## Conflicts of Interest

The authors declare no conflicts of interest.

## Author Contributions

Zhongyong Zhang, Zhongqian Han, and Qianqian Jin carried out the experiments and manuscript writing. Hongmin Zhao and Yingkai Liu provided experimental help. Qianqian Jin, Meng Wang, Zhongqian Han, and Jin Wu performed data analysis and result interpretation. Shuquan Lv provided ideas and performed manuscript review & editing. Xiaoyun Wang, Wei Chen, and Qingjin Wang performed data analysis. Hailong Bai performed data analysis. Yuansong Wang supervised the experiments. All authors contributed to the article. Zhongyong Zhang and Qianqian Jin are co‐first authors.

## Funding

This study was supported by The China Medical Association of Minorities, 2020ZY118‐580101 and the Hebei Administration of Traditional Chinese Medicine, 2020515.

## Supporting information


**Supporting Information** Additional supporting information can be found online in the Supporting Information section. The details of materials, reagents can be found in supplementary materials. Table S1: Elution gradient table. Figure S1: The chemical profiles of QGMS using UPLC‐MS. Figure S2: The effects of QGMS on insulin resistance. Figure S3: Statistical results of intake of mice during the experiment period.

## Data Availability

Data will be made available on request.
