# Supplementary material for: Qinggan Mingshi Granules Inhibited Ferroptosis to Treat Diabetic Retinopathy in Mice Through NRF2/GPX4 Axis
Source: J Diabetes Res. 2026 Jan 21;2026:9978155. doi: 10.1155/jdr/9978155 (PMC12824454; doi:10.1155/jdr/9978155)
Supplement: Supplementary file 1 — Supporting Information Additional supporting information can be found online in the Supporting Information section. The details of materials, reagents can be found in supplementary materials. Table S1: Elution gradient table. Figure S1: The chemical profiles of QGMS using UPLC‐MS. Figure S2: The effects of QGMS on insulin resistance. Figure S3: Statistical results of intake of mice during the experiment period. [file JDR-2026-9978155-s001.docx]

**1 Reagents** **and assay kits**

Streptozotocin (S17049), calcium dobesilate (S63094), Ferrostatin-1 (S81461), and ML385 (S86700) were purchased from Shanghai yuanye Bio-Technology Co., Ltd. Assay kits for total protein, ROS, total iron, malondialdehyde (MDA) were purchased from Nanjing Jiancheng Biological Engineering Institute. Terminal deoxynucleotidyl transferase dUTP nick end labeling (TUNEL) staining assay kit was purchased from Beyotime Biotechnology. ELISA kits for4-hydroxynonenal was purchased from Shanghai Enzyme-linked Biotechnology Co., Ltd. Primary antibodies for NRF2 (16396-1-AP), SLC7A11 (26864-1-AP), FTL (10727-1-AP), GPX4 (67763-1-lg), beta-actin (20536-1-AP), and secondary antibody goat anti-rabbit IgG H&L (SA00001-2), anti-mouse IgG H&L (SA00001-1) were purchased from Proteintech.

**2 UPLC-MS analysis of** **QGMS**

QGMS was obtained from Cangzhou Hospital of Integrated Traditional Chinese Medicine and Western Medicine. Ultra performance liquid chromatography (UPLC) coupled with mass spectrometer (MS) was conducted as the quality control of QGMS.

**2.1 Sample preparation and extraction**

2.1.1 Take a certain mass of powder, add methanol, ultrasonic dissolution for 1 h.

2.1.2 Take 100 μL solution and dilute it with 50 % methanol to 100 μg / mL.

2.1.3 The centrifuge tube was placed in a low-temperature centrifuge and centrifuged at 4 °C, 12000 rpm for 5 min.

2.1.4 A total of 100 μL supernatant was placed in the injection bottle for mass spectrometry detection.

**2.2 HPLC Conditions**

Column: Waters UPLC HSS T3 (1.8 μm, 2.1 mm × 100 mm)

Mobile Phase: A phase (water + 0.1% formic acid), B phase (methanol)

Gradient Elution: Refer to Table S1

Flow Rate: 0.3 mL/min

Injection Volume: 10.0 µL

Column Temperature: 40°C

**Table S1** Elution gradient table

| Time（min） | Mobile Phase | |
| --- | --- | --- |
|  | A（v%） | B（v%） |
| 0 | 98 | 2 |
| 1.0 | 98 | 2 |
| 10.0 | 0 | 100 |
| 14.0 | 0 | 100 |
| 14.1 | 98 | 2 |
| 16.0 | 98 | 2 |

**2.3 MS Conditions**

Mass spectrometric analysis was performed using a quadrupole orbitrap mass spectrometer equipped with a heated electrospray ionization source (Q Exactive™). The ion source voltages for both positive and negative ions were set at 3.7 kV and 3.5 kV, respectively. The capillary temperature was maintained at 320°C. Sheath gas pressure was set at 30 psi, and auxiliary gas pressure was set at 10 psi. The solvent heated-evaporative gas temperature was 300°C. Both sheath and auxiliary gases were nitrogen, while the collision gas was also nitrogen with a pressure of 1.5 mTorr. For the primary full scan, the parameters were as follows: resolution 70000, automatic gain control target 1×10^6^, maximum injection time 50 ms, mass-to-charge ratio scan range 100–1500. Mass axis calibration was achieved using an external standard method with a mass error of 5 ppm. Positive ion calibration standards were 74.09643, 83.06037, 195.08465, 262.63612, 524.26496, and 1022.00341; negative ion calibration standards were 91.00368, 96.96010, 112.98559, 265.14790, 514.28440, and 1080.00999. Metabolite identification utilized a dd-MS2 scan mode (data-dependent scan mode) with the following parameters: resolution 17500, automatic gain control target 1×10^5^, maximum injection time 50 ms, up to 10 ions in the second-level fragmentation (dynamically excluded), mass isolation window 2, collision energy 30 V, intensity threshold 1×10^5^. The Thermo Xcalibur 2.2 SP1.48 software controlled the liquid chromatography-mass spectrometry system and facilitated data acquisition.

**2.4 UPLC-MS analysis results of** **QGMS decoction**

We found that Alisol B 23-acetate, Ginsenoside Rg1, Ginsenoside Re, Ginsenoside Rb1, Catalpol, Astragaloside A, Calycosin-7-O-beta-D-glucoside, Puerarin, Aloeemodin, Rhein, Emodin, and Chrysophanic acid widely existed in QGMS (**Figure S1**).

a


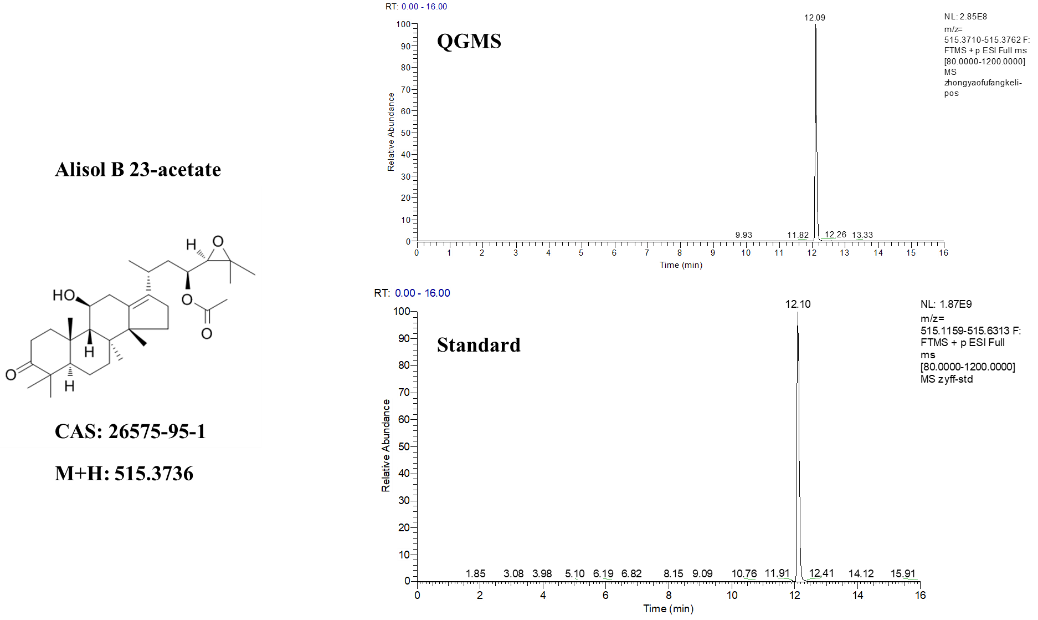


b


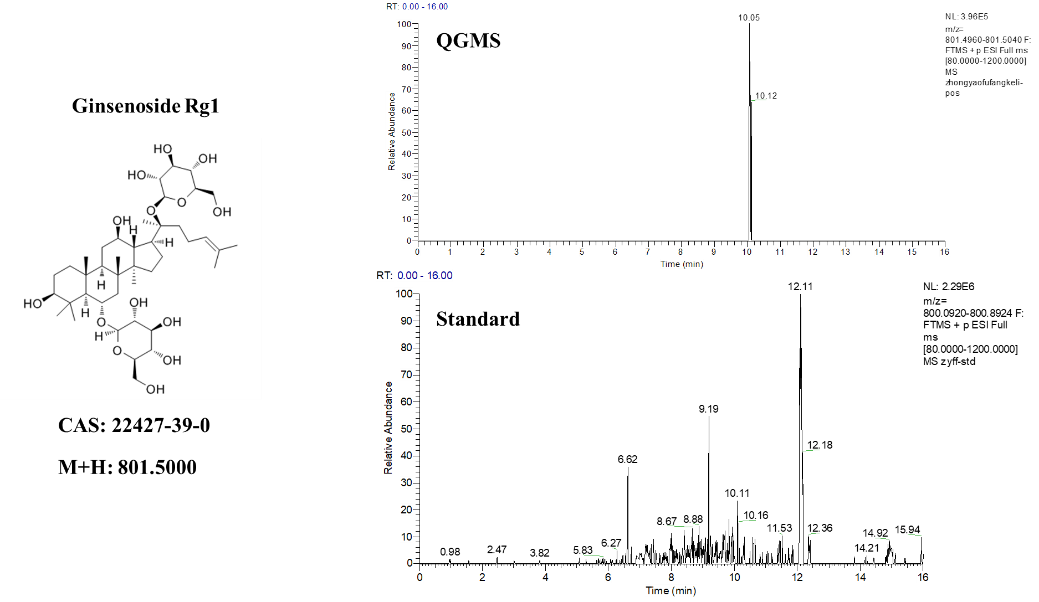


c


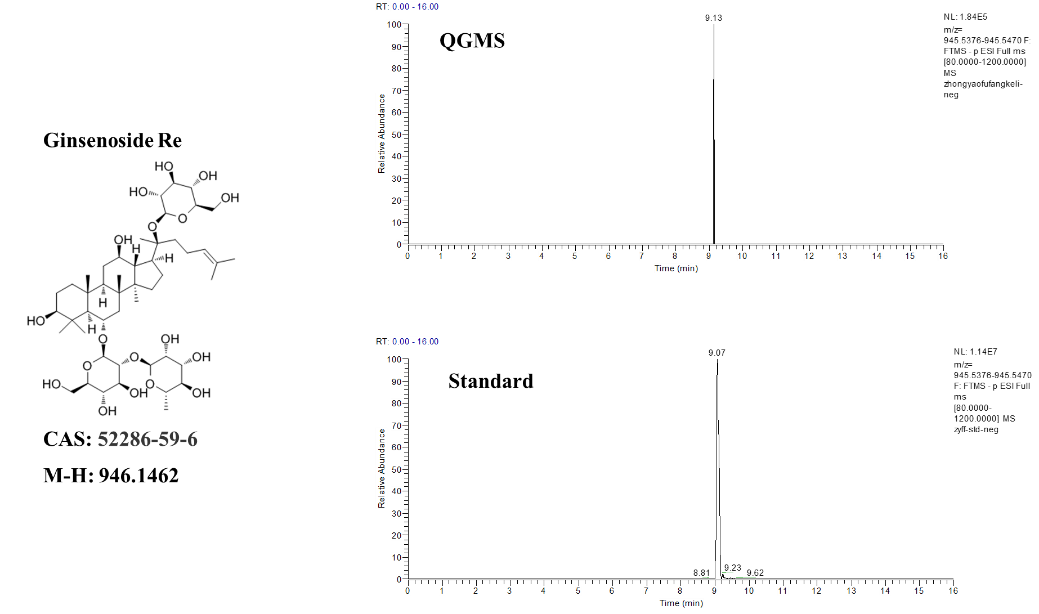


d


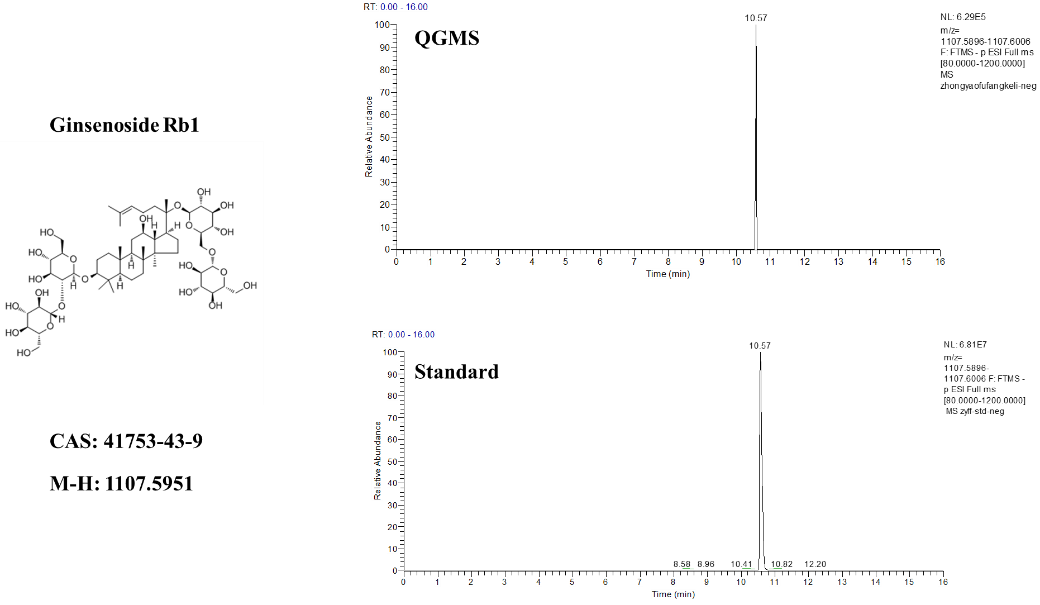


e


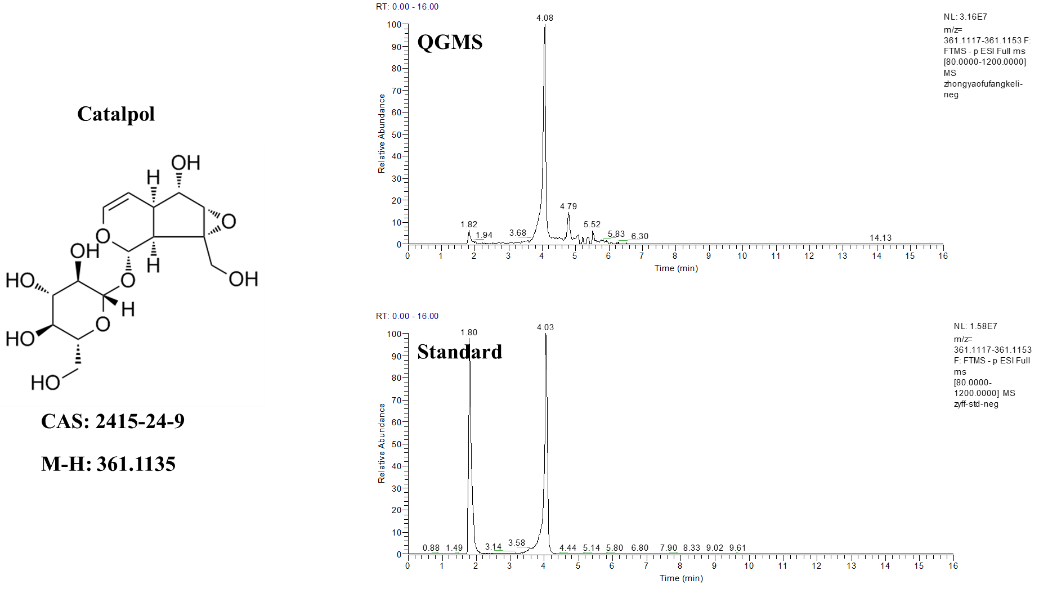


f


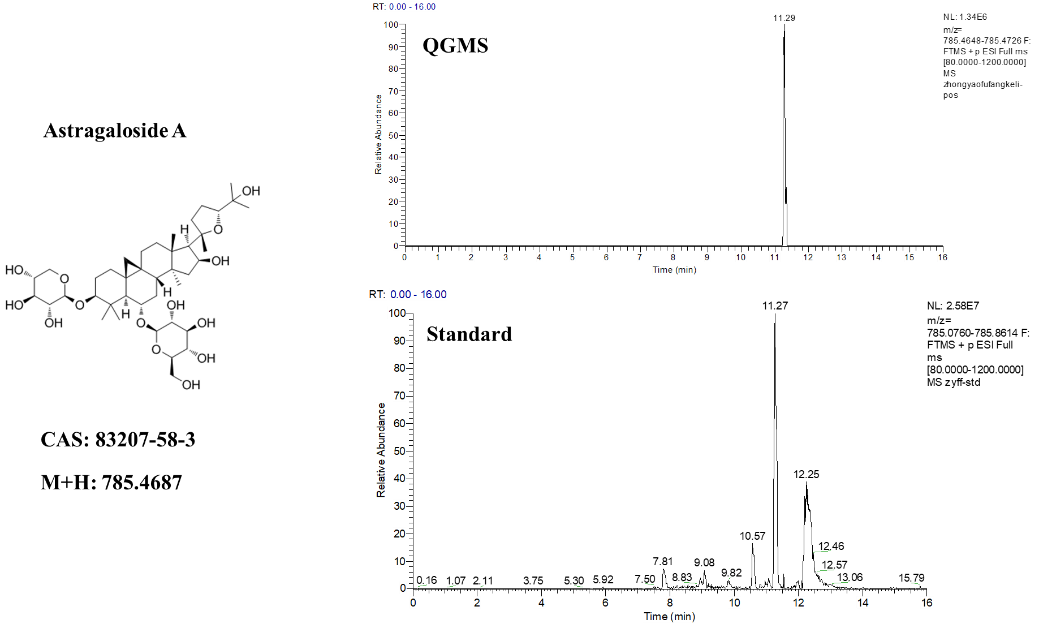


g


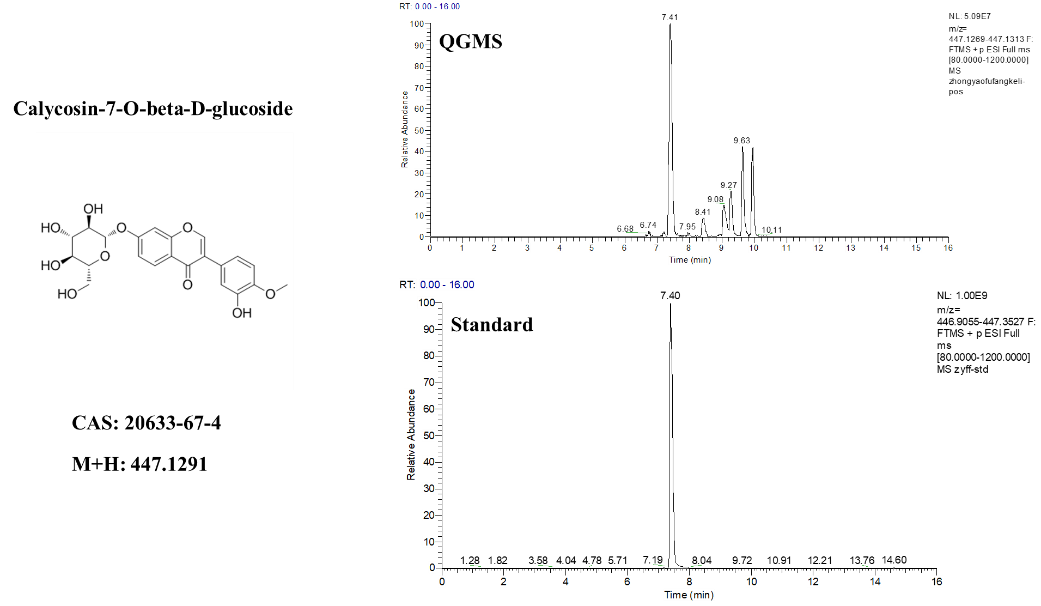


h


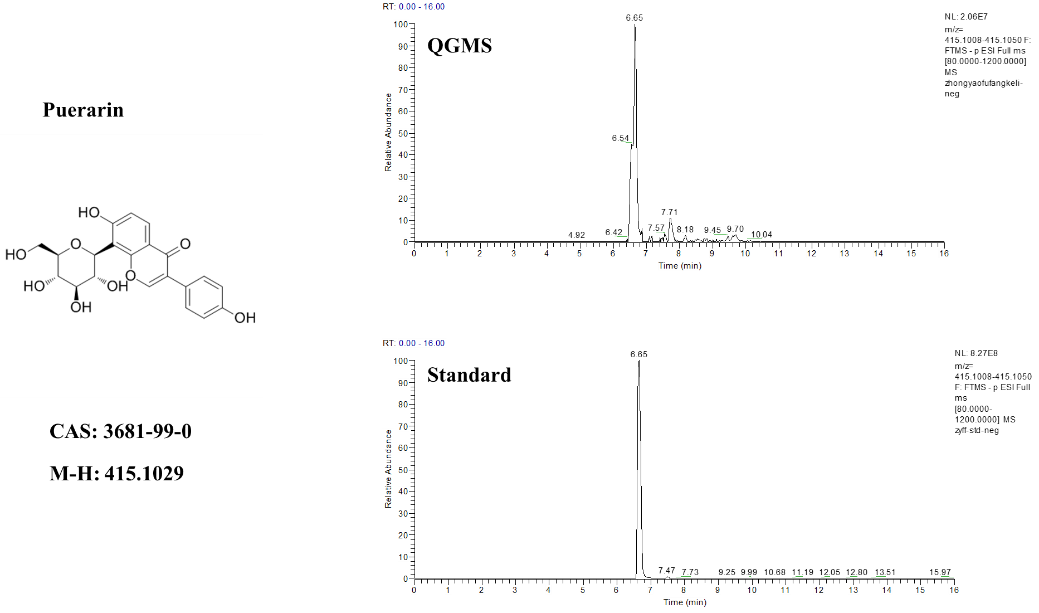


i


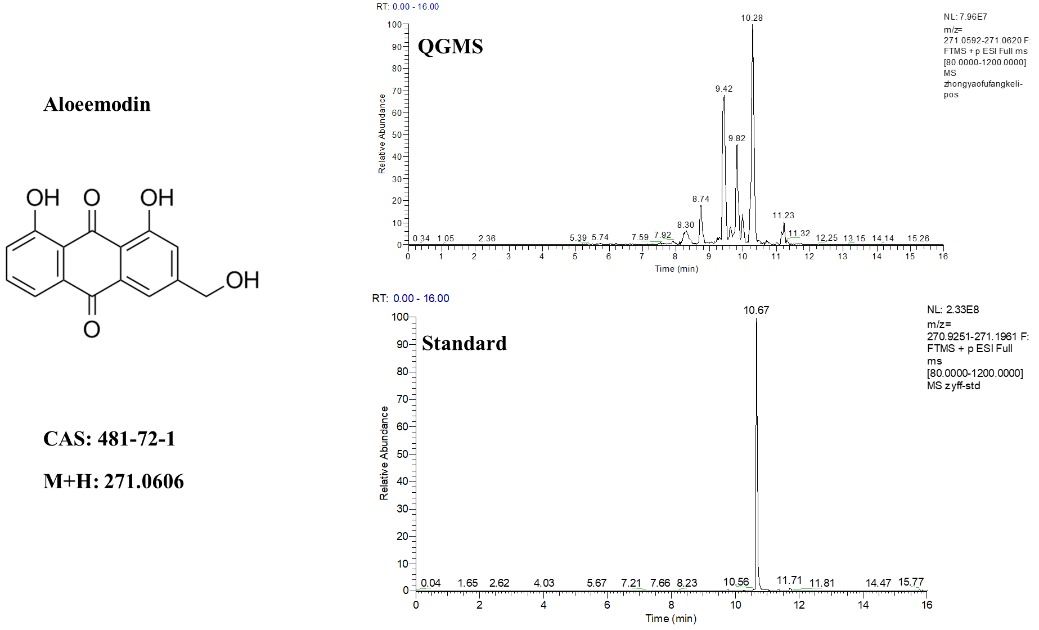


j


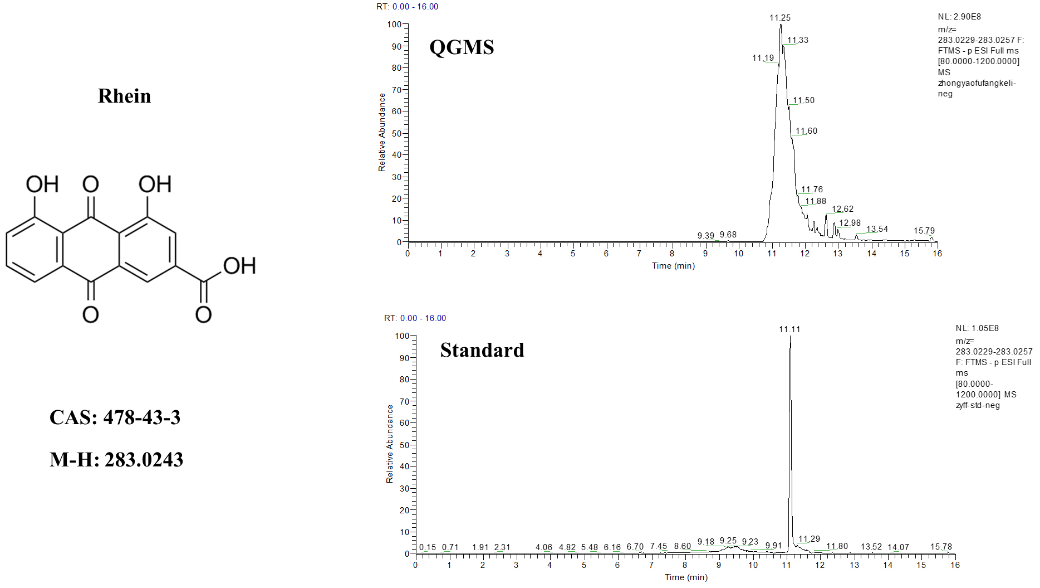


k


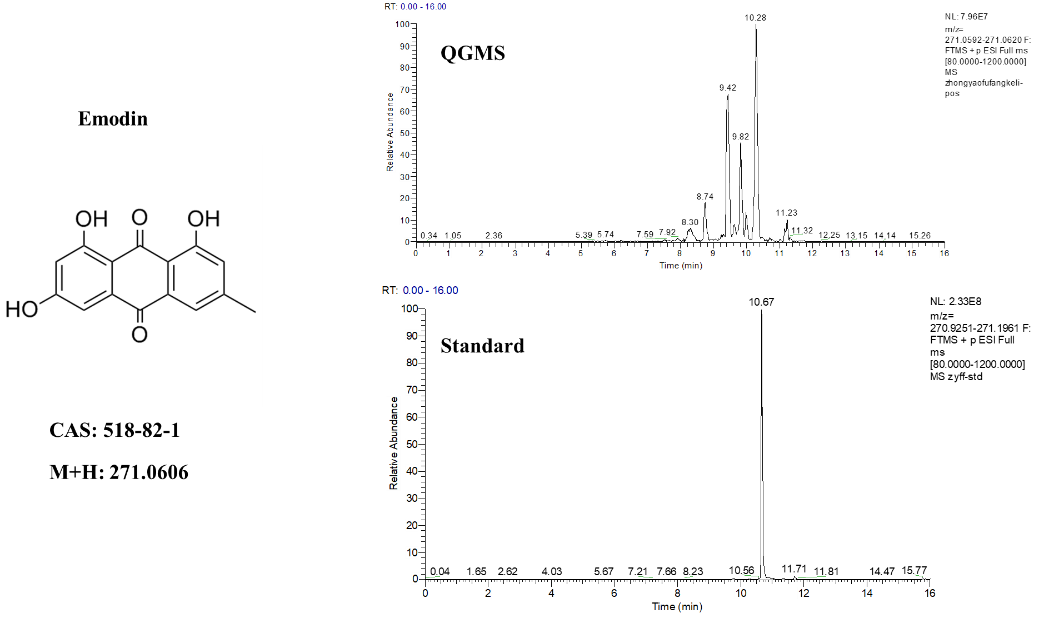


l


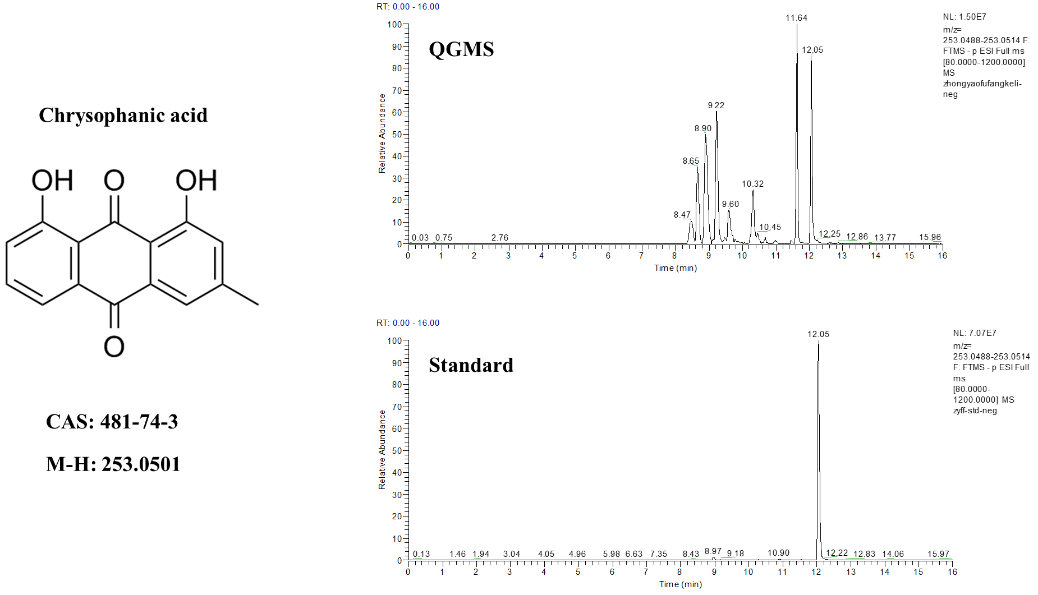


**Figure S1:** The chemical profiles of QGMS using UPLC-MS.


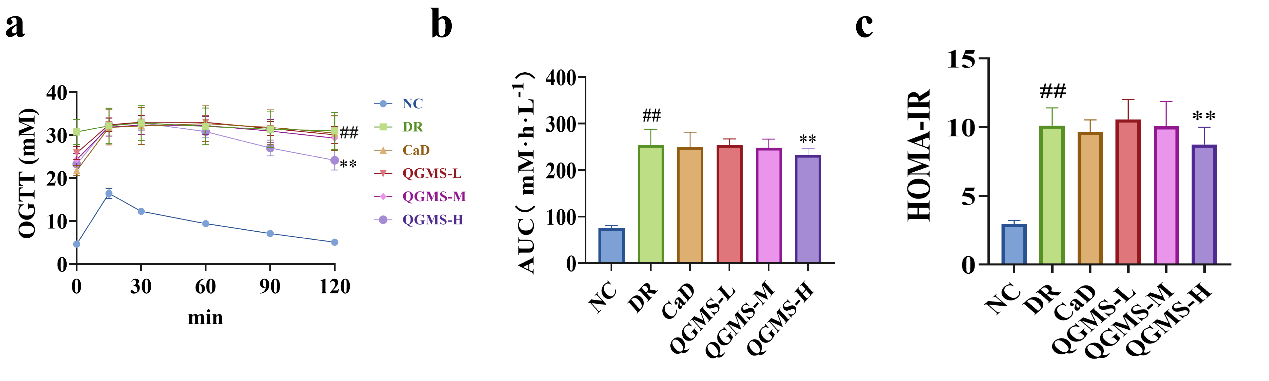


**Figure S2:** The effects of QGMS on insulin resistance.

(a) OGTT; (b) AUC; (c) HOMA-IR. ##: *p* < 0.01 compared with NC; **: *p* < 0.01 compared with DR group.


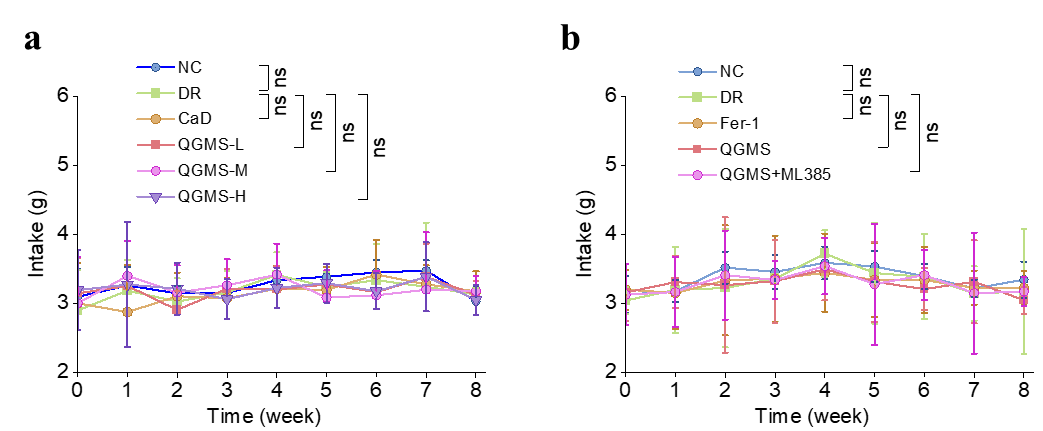


**Figure S3**: Statistical results of intake of mice during the experiment period. n=20 per group. ns: no significance.
